# Self-Oscillating Liquid Gating Membranes with Periodic Gas Transport

**DOI:** 10.3390/membranes12070642

**Published:** 2022-06-23

**Authors:** Xue Xu, Jing Liu, Min Cao, Jian Zhang, Xinlu Huang, Xu Hou

**Affiliations:** 1 State Key Laboratory of Physical Chemistry of Solid Surfaces, College of Chemistry and Chemical Engineering, Xiamen University, Xiamen 361005, China; xuxue@stu.xmu.edu.cn (X.X.); jingliu11@stu.xmu.edu.cn (J.L.); 20420201151636@stu.xmu.edu.cn (M.C.); jianzhangchem@stu.xmu.edu.cn (J.Z.); 36520211151757@stu.xmu.edu.cn (X.H.); 2 Fujian Provincial Key Laboratory for Soft Functional Materials Research, Research Institute for Biomimetics and Soft Matter, College of Physical Science and Technology, Xiamen University, Xiamen 361005, China; 3 Innovation Laboratory for Sciences and Technologies of Energy Materials of Fujian Province (IKKEM), Xiamen 361005, China

**Keywords:** liquid gating membrane, self-oscillating behavior, gas–liquid interface, critical pressure, periodic gas transport

## Abstract

Liquid gating membranes with molecular-level smooth liquid lining layers break through the limitations of traditional porous membrane materials in gas transport control. Owing to the stable, self-healing, and reconfigurable properties, liquid gating membranes have shown wide application prospects in microfluidics, intelligent valves, chemical reactions, and beyond. Here, we develop a periodic gas transport control system based on the self-oscillating liquid gating membrane. Under continuous gas injection, the gas–liquid interface is reversibly deformed, enabling self-oscillating behavior for discontinuous and periodic gas transport without the need for any complex external changes to the original system. Meanwhile, our experimental analysis reveals that the periodic time and periodic gas release in the system can be regulated. Based on the cycle stability of the system, we further demonstrate the controllability of the system for periodic droplet manipulation in microfluidics. Looking forward, it will offer new opportunities for various applications, such as pneumatic robots, gas-involved chemical reactions, droplet microfluidics, and beyond.

## 1. Introduction

Benefiting from utilizing the capillary-stabilized liquid as a dynamical and reconfigurable gate, liquid gating membranes have shown outstanding performance for fluid transport control in response to the pressure gradient [1]. The transport gases/liquids will deform the interface of the pore-filling gating liquid to enter the pore under the specific critical pressures (*P*_c_). Particularly, for traditional solid porous membrane materials, gas transport behavior is uncontrolled and even occurs at zero pressure in the gas-solid interfaces. While in the liquid gating membrane, the gas-solid interfaces are transformed into the gas/liquid/solid interfaces [2]. It is necessary to overcome the gas–liquid interface tension for gas permeation, resulting in the *P*_c_ of gas transport being greater than zero. Therefore, the liquid gating membrane has flexible and stable switching functions in gas transport [3], which enables liquid gating technology to facilitate the development of many intelligent applications, such as chemical reactions [4], multiphase separations [5], and chemical detections [6].

In general, the pressure threshold in liquid gating membranes for gas transport is mainly affected by the properties of the gating liquid, such as viscosity, chemical composition, concentration, and surface tension [7]. Recent works have shown some progress toward the dynamic interface research in gas transport control. For example, when the analyte cation was added to the gating liquid, due to the dipole-induced interfacial molecular reconfiguration in the dynamic gas-liquid interface, the transmembrane critical pressure was decreased. The released gas made the marker droplet move, which could be used as a detection signal information, and open up new avenues for chemical detection [6]. In addition, based on the reversible photoisomerization of molecular photoswitches induced a variation of the solid–liquid interaction between the solid porous substrate and the gating liquid, a light-responsive gas valve with controllable gas transport under the external stimuli of UV light has been developed [8]. More recently, a liquid gating CO_2_ chemical response valve has performed self-adaptive switching behaviors with transporting gases mixed with CO_2_, utilizing the neutral superamphiphilic compound in the gating liquid that was protonated by CO_2_. Compared with other injected gases, such as Ar, N_2_, or O_2_, when CO_2_ was transported, a higher transmembrane pressure was required to open the system. Thus, the system could be closed at low applied pressures [9]. Although much progress in the demonstration of various controls on gas transport has been made in multifarious liquid gating membranes, gas transportation in previous works is uninterrupted, and usually relies on the changes in the external regulatory environment, including the modulation of gas–liquid interface properties, external stimuli, or injection sources to drive them to perform reversibly closed/open states. The liquid gating membrane itself with self-oscillating switching behavior for discontinuous and periodic gas transport has not been realized, which is of great significance in practical applications without any complex external regulations.

Herein, we design a self-oscillating liquid gating membrane (SOLGM) for intermittent and periodic gas transport. Under continuous gas injection, the pressure of the system increases, accompanied by the deformation of gas–liquid interfaces. After rising to the *P*_c_, the rapid release of gas leads to decreased pressure and reversible reconfiguration of the gating liquid. This work successfully realizes the self-oscillating behavior with periodic closed/open states and periodic gas transport control without any complex external operational variations. To demonstrate the tunability of the self-oscillation behavior, we altered the periodic time of interface deformation and achieved the regulated periodic release in gas transport by applying different constant flow rates. Moreover, periodic gas release drives droplet motion, which demonstrates the excellent performance of the system for periodic gas transport in microfluidic droplet systems. We believe that this system is an important step toward fuel cells, gas valves, periodic fluid transport, etc.

## 2. Materials and Methods

### 2.1. Materials and Chemicals

Polytetrafluoroethylene (PTFE) membranes were purchased from Jincheng Plastic online store (Guangdong, China). The stainless steel mesh (SSM, 316 L) was purchased from Anping Tianhong Metal Mesh Factory (Hebei, China). Stainless steel sheets (316 L) were provided by Dongtai Penglian Stainless-steel Products Co., Ltd. (Dongtai, China). Ethanol was purchased from Sinopharm Chemical Reagent Co., Ltd. (Shanghai, China). Silicone oil with a viscosity of 500 cP (Silicone oil-500, nonvolatility) was purchased from Aladdin Industrial Co., Ltd. (Shenzhen, China), without any further treatment. Eykosi magic waterproof and antifouling spray were purchased from Taobao. Air was used as the injected gas. The magenta filling ink was provided by TIANSE Stationery Co., Ltd. (Guangzhou, China). Deionized water (18.2 MΩ·cm) was obtained from Mili-Q Integral 3 System.

### 2.2. Fabrication of SOLGM

In the experiment, SOLGM was designed as single-pore systems or porous systems. Silicone oil-500 was used for the gating liquid.

Single-pore systems consist of the single-pore membrane and the gating liquid. Single-pore membranes (diameter of 25 mm) were fabricated from flat PTFE membranes by Femtosecond Laser Cutting at the Technical Institute of Physics and Chemistry, Chinese Academy of Sciences. Firstly, the AutoCAD 2021 software (Autodesk, San Rafael, CA, USA) was used to design and draw the pore size diagrams of the system. Secondly, by adjusting the appropriate cutting speed and laser intensity, the pore size of the single-pore can be controlled. Then the membranes were moved to the optical microscope for observation to measure the actual pore sizes of single-pore membranes. After that, the membranes were cleaned by ultrasonic for 10 min in the sequence of ethanol and deionized water, respectively. After being dried, they were prepared by dropping ~10 µL/cm^2^ gating liquid on the surfaces of single-pore membranes, and uniform coverage was achieved after a while.

Porous systems consist of a porous membrane and a gating liquid. Porous membranes (diameter of 25 mm) adopt the SSM. The SSM was cleaned through ultrasonic in ethanol and deionized water in sequence for 10 min to remove surface contaminants. After being dried, the ~60 µL/cm^2^ gating liquid with a volume of 60 µL was added dropwise to infiltrate the porous membrane fully.

### 2.3. Characterizations

The self-oscillating behavior of SOLGM was characterized by a TH4-200 microscope (Olympus, Tokyo, Japan). The photographs of the demonstration of SOLGM for periodic gas transport control and videos were taken by a digital camera (Nikon D750).

### 2.4. Transmembrane Pressure Measurements

The gas transport behavior of SOLGM was characterized by measuring the transmembrane pressure during the gas flow. Both two sides of SOLGM were provided with a gas inlet and outlet. Air was used for gas transport. The difference in the transmembrane pressure between the gas inlet and outlet of the system was measured by a wet/wet current output differential pressure transmitter (PX154-025DI, Omega, Stamford, CT, USA) purchased from OMEGA Engineering Inc. (Stamford, CT, USA). In all experiments, a Harvard Apparatus PHD ULTRA Syringe Pump with a 50 mL syringe was used for gas injection at constant flow rates of 0.05, 0.1, 0.25, 0.5, and 1 mL/min, respectively. Considering the uncertainties in the experiment (including the fluidity of the gas, the interaction of the gas–liquid interface, and the accuracy of the instrument), in order to ensure the reliability and rigor of the experiment, the values of all experiments used were averages of at least three measurements and at room temperature.

### 2.5. Contact Angle Measurements

Contact angles (CA) of silicone oil-500 were measured by the sessile drop method on an optical angle measuring instrument (OCA100, Dataphysics, Stuttgart, Germany) at room temperature on the surface of PTFE membranes and SSMs. It was worth emphasizing that we prevented the influence of the pore structure on the wettability, the same membrane material without pores was used for measurement. The dosing volume of silicone oil 500 was 5 µL at a dosing rate of 1 µL/s and photos were taken after 30 s. The values of contact angles were averages of at least three independent measurements.

## 3. Results and Discussions

### 3.1. Operation Mechanism of SOLGM

In living things, the alveoli are full of micron-sized pores filled with liquids, realizing gas transport control between alveoli and blood tissues when driven by pressure [10]. The liquid gating membrane was inspired by such natural phenomena [11,12] that use liquid-filled pores of solid membranes to control fluid transport. Under the action of the capillary force, liquid can be stabilized in the micron pores. Therefore, the pores can be reversibly sealed in a closed state and rapidly reconfigured under pressure-driven to generate an open state and liquid-lined pathway. The system shows a flexible and stable switching performance, which breaks through the limitations of traditional solid membrane materials in gas transport control [13,14,15,16].

**Figure 1:**
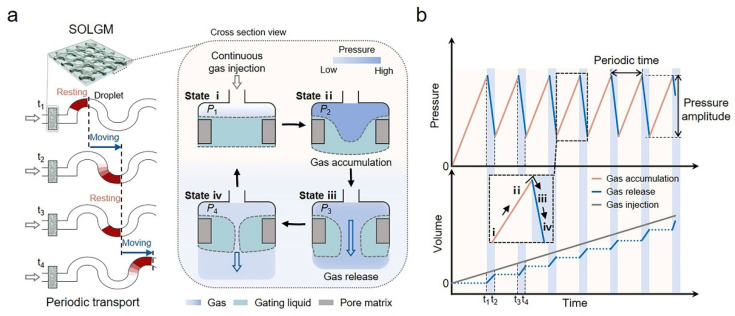
Schematics of liquid gating self-oscillating membrane (SOLGM) with periodic gas transport. (**a**) The compressible gas was continuously injected into membrane pores infiltrated with the incompressible gating liquid and released through pressure-induced deformation of the gas–liquid interface. The gating liquid reversibly infiltrated the membrane pores due to the decrease in pressure in the open state. Then, the system would be in a closed state. Therefore, periodic gas transport control can be achieved by self-oscillating closed/open states. (**b**) Relationships among pressure, volume, and time in SOLGM.

Figure 1 provides an illustration of the operation mechanism of SOLGM for periodic gas transport. A gas–liquid interface was formed at the junction of the gating liquid and the transport gas. With the continuous gas injection, the gas accumulated and compressed [17], and the applied pressure (∆*P*) increased, resulting in gas–liquid interface deformation and an unbalanced state. Gas–liquid interfaces are deformable as follows: they can freely change their shape to minimize their surface energy [15], in the same way as the transition from initial state i to state ii in Figure 1a (*P*_1_ < *P*_2_). When the ∆*P* increased to the *P*_c_, SOLGM was in the open state (state iii and state iv in Figure 1a). Meanwhile, the gas was released instantaneously, and the pressure decreased (*P*_1_ < *P*_4_ < *P*_3_ < *P*_2_, Figure 1a). Due to the effect of capillary force, which brings the system back to the initial closed state (state i in Figure 1a). In short, SOLGM mainly undergoes four state transitions in one periodic time, from state i to state ii, state iii, and state iv, which change periodically under the drive of the ∆*P* and interfacial tension. ∆*P* that must be overcome is the Laplace pressure for gas transport [18].

(1) $$\Delta P=\frac{4\gamma_{g-l}}{d}$$

where $\gamma_{g-l}\;$ is the gas–liquid surface tension, and d is the average effective pore size.

Therefore, this strategy allows SOLGM to perform periodic gas transport control under continuous gas injection (Figure 1a). A channel with SOLGM was designed. When the time was at t_1_, the droplet was in the original position. With the gas injection, the pressure of the system increased to the *P*_c_, the system would be opened. After that, the instantaneous gas release made the position movement of the droplet (t_2_ in Figure 1a), resulting in a drop in the system pressure, and the gas–liquid interface was reconstructed. The system was in a closed state from t_2_ to t_3_, and the droplet kept a resting state. Then, the ∆*P* rose to the *P*_c_ again, the system was in the open state and the droplet would be moving from the position of t_3_ to t_4_. The periodic motion of the droplet in the channel demonstrates gas release behavior can be controlled and periodic. To better illustrate SOLGM with periodic gas transport behavior, Figure 1b shows the relationship between pressure, volume, and time in SOLGM. In addition, periodic time refers to the time required for a complete process of SOLGM to go from the open state to the closed state or from the closed state to the open state. Pressure amplitude is called the range of pressure variation of SOLGM in the open state. No additional parameters need to be regulated. With the increase in gas volume, the system can spontaneously generate gas relief and realize self-oscillating behavior with the closed/open state control, which can achieve periodic gas transport.

### 3.2. Characterization of Self-Oscillating Behavior with Periodic Gas Transport

**Figure 2:**
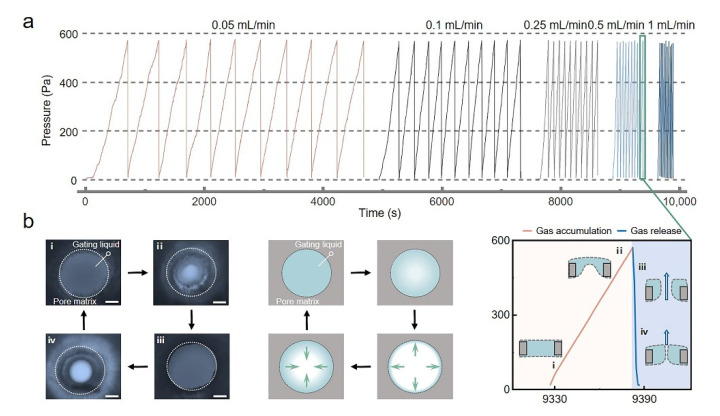
Characterization of the self-oscillating behavior with the periodic closed/open states. (**a**) Evolution as a function of time of SOLGM (Here, SOLGM is a single-pore, the pore size is 220 µm) with different flow rates of pressure. (**b**) Left: optical microscopy images of the four states of SOLGM and schematic diagram of the four states during one periodic time (state i: the initial closed state, state ii: the transition state from closed state to open state, state iii: the open state, state iv: the recovery state from open state to closed state). Right: local enlargement of the pressure and time relationship of SOLGM in one periodic time at a flow rate of 0.5 mL/min, and schematic of the state diagrams. Scale bars: 100 µm.

The self-oscillating behavior of SOLGM is shown in Figure 2. The system consisted of a single-pore membrane with a pore size of 220 µm and a gating liquid. Gas was injected at constant flow rates of 0.05, 0.1, 0.25, 0.5, and 1 mL/min, respectively. With the flow rate increasing (Figure 2a), the periodic time decreased gradually, and ∆*P* was not affected. The range of ∆*P* is only related to the nature of SOLGM itself, such as the gating liquid or pore size, and is not affected by the change in flow rate.

Meanwhile, we observed the self-oscillating closed/open states with periodic gas transport of SOLGM under an optical microscope (Supplementary Materials Video S1). As an example, the system performs self-oscillating behavior at a flow rate of 0.5 mL/min at one periodic time. SOLGM was transformed from initial state i to state ii (Figure 2b) by pressure-driven deformation of the gas–liquid interface under gas injection, and the system was still in a closed state. When ∆*P* reached *P*_c_, the system was in the open state, and gas was released instantaneously. Then the system was in state iii. After the pressure gradually decreased to state iv, the gating liquid was reconstructed and returned to state i. Therefore, we further designed single-pore systems and porous systems to study the self-oscillating behavior of SOLGM for periodic gas transport.

### 3.3. Design Strategy and Parameter Regulation of SOLGM

#### 3.3.1. Stable Single-Pore Systems

**Figure 3:**
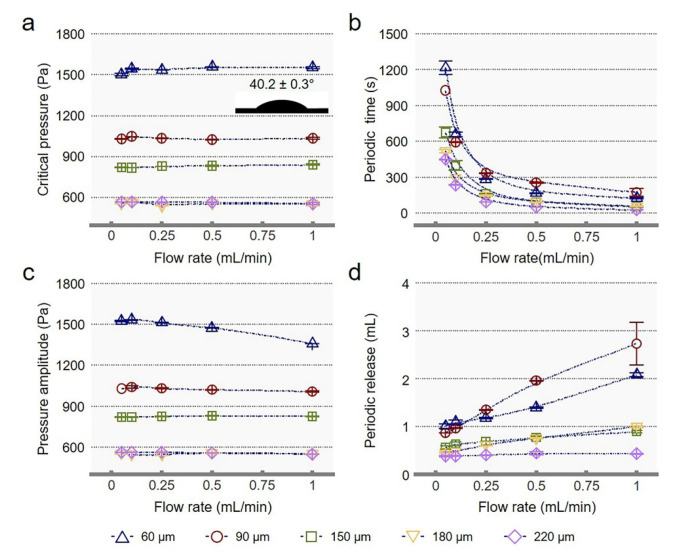
Design and regulation of self-oscillating behavior of the single-pore. Evolution as a function of the flow rate of the single-pore system with different pore sizes of (**a**) critical pressure, (**b**) periodic time, (**c**) pressure amplitude, and (**d**) periodic release. The gating liquid was measured on polytetrafluoroethylene (PTFE) membranes to characterize the wettability of single-pore systems.

Figure 3 shows the design and regulation of the self-oscillating behavior of single-pore systems. The single-pore system consists of a single-pore membrane and a gating liquid. Single-pore membranes were made from flat PTFE membranes by laser cutting methods. According to the adjustment of laser cutting intensity and cutting speed, we mainly fabricated single-pore membranes with pore sizes of 60, 90, 150, 180, and 220 µm. To achieve better self-oscillating behavior, we designed a stable single-pore system. The following requirements must be met: (1) the gating liquid must have an affinity for the single-pore membrane and can completely infiltrate the single-pore membrane; (2) there is no mutual solubility between the gating liquid and the transport fluid; (3) the affinity between the transport fluid and the single-pore membrane must be lower than that between the gating liquid and the single-pore membrane, in case the transport fluid replaces the gating liquid in the transportation process [19]. But for gas transport, as long as the gating liquid is very affinitive to the membrane, the system can be very stable. The wettability of gating liquid on single-pore membranes was measured by the sessile drop method. The CA of the gating liquid is shown in Figure 3. The gating liquid has a CA value of 40.2 ± 0.3°, indicating that the gating liquid is very compatible with the single-pore membrane [20]. However, the *P*_c_ (Figure 3a) and pressure amplitude (Figure 3c) of different pore sizes are almost constant with the increase in flow rate. However, the *P*_c_ and pressure amplitude of single-pores decrease gradually with the increase of pore size. The Laplace equation shows that the pressure is inversely proportional to the average effective pore size. The larger the average pore size, the smaller the pressure.

Meanwhile, with the increase in flow rate, the periodic time gradually decreases (Figure 3b). Single-pore systems of the same pore size have a specific *P*_c_, and the pressure is a change caused by the compression of gas injection. The lower the flow rate of gas injection, the more time it takes for the ∆*P* to rise to the *P*_c_. Moreover, with the increase in pore size, the periodic time and periodic release of the system for gas transport show a decreasing trend as a whole under the same flow rate. Like the above, because of the larger pore size, the smaller pressure, and the less time required to reach the *P*_c_. The smaller pressure means the smaller volume of gas accumulation, and therefore, the smaller periodic release. In addition, the relationship between the periodic gas release of the single-pore system with the same pore size and the flow rate is proportional; the larger the flow rate, the greater the periodic release (Figure 3d). As the flow rate increases, the volume of gas released per unit time will also increase in the open state. Based on the above results, the single-pore system can be used as a stable system for periodic gas transport. Changing the gas injection flow rate can realize the gas transport control with variable periodic time and release quantity. Therefore, quantitative and periodic gas transport control devices can be designed.

#### 3.3.2. Stable Porous Systems

**Figure 4:**
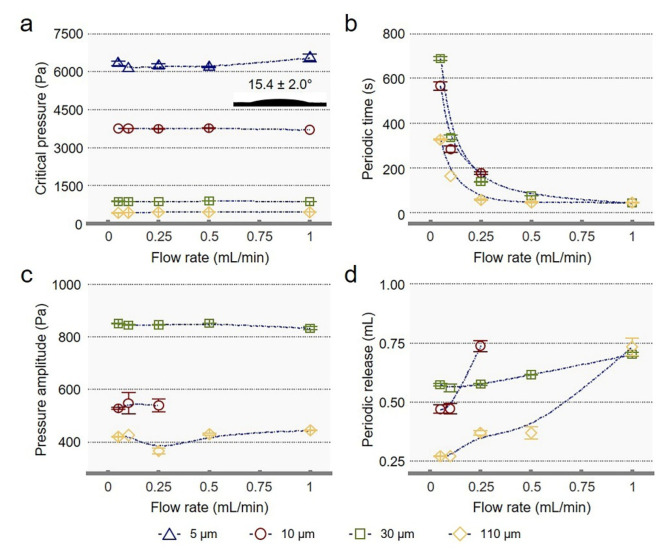
Periodic transport in porous systems. Evolution as a function of the flow rate of SOLGM with different pore sizes of (**a**) critical pressure, (**b**) periodic time, (**c**) pressure amplitude, and (**d**) periodic release. The gating liquid was measured on stainless steel meshes to characterize the wettability of porous systems.

In order to verify the universality of the self-oscillating behavior in SOLGM for periodic gas transport, reasonable adjustments were made, and further studies would be carried out in porous systems. Here, the porous system consists of a porous membrane and a gating liquid, and the porous membrane uses SSMs. As mentioned above, in single-pore systems for gas transport, on the condition that the gating liquid is very affinitive to the membrane, SOLGM can be very stable. The wettability of gating liquid on porous systems was measured by the sessile drop method. The CA of the gating liquid is shown in Figure 4a. The gating liquid has a CA value of 15.4 ± 2.0°, indicating that the gating liquid is very compatible with the porous membranes. We selected SSMs with pore sizes of 5, 10, 30, and 110 µm to construct the porous system. It is worth noting that the pore size of SSMs we have converted is based on the mesh parameters of SSMs, not the actual pore size.

For the porous systems, the change of the flow rate also cannot affect the *P*_c_ of the system due to the properties of the system itself. The periodic time will decrease as the flow rate of the porous membranes increases (Figure 4b). Meanwhile, the relationship between *P*_c_, periodic amplitude (Figure 4c), periodic time, and pore size of porous systems is also consistent with single-pore systems. Notably, with the increase in pore size, the changes in periodic release (Figure 4d) are not fully consistent with single-pore systems. This is mainly due to abrupt changes in the data for the porous system with a pore size of 10 µm under the flow rate of 0.25 mL/min. We consider that since the SSM with a pore size of 10 µm is a braided twist structure [21], the influence of its structure increases the error. Moreover, neither the system with a pore size of 5 µm at any flow rate in the experiment nor the system with a pore size of 10 µm at a flow rate of 0.5 mL/min and 1 mL/min shows self-oscillating gas release with periodic closed/open states. The results indicate that the permeability is mainly based on the pore-flow model [22,23,24], and the self-oscillating behavior would be affected by pore size or flow rate.

### 3.4. Demonstration of SOLGM for Periodic Gas Transport in Droplet Microfluidics

After systematic exploration of the periodic gas release inside the SOLGM, we further demonstrated the functionality for periodic gas transport (Supplementary Materials Video S2). Referring to the devices of droplet microfluidics [25], we designed a channel with a cross-section of the entrance of a rectangle with a length of 4 mm and a width of 5 mm. Air was used as the transport gas, and droplets (injected by pipette before the connection with the self-oscillating system) with a volume of 200 µL were used to indicate the trajectory of the movement. We utilized a porous system (pore size of 30 µm) at a constant gas injection flow rate of 1 mL/min to demonstrate periodic gas transport. In order not to consider the influence of friction resistance on the system, the device was hydrophobized (the channel was sprayed with eykosi magic waterproof and antifouling spray and dried naturally).

**Figure 5:**
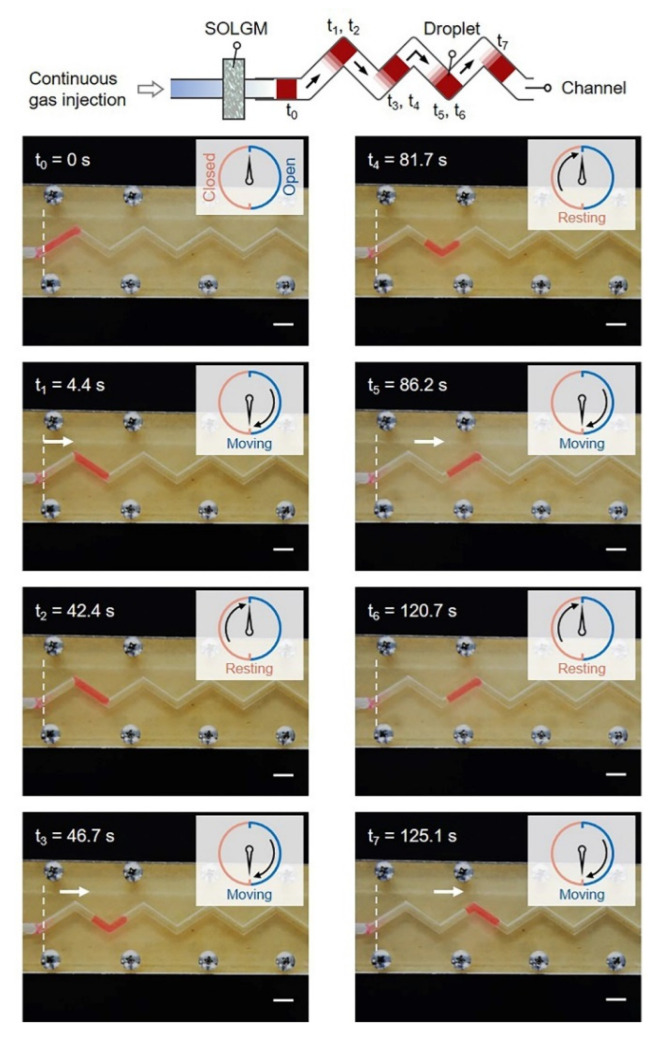
Demonstration of SOLGM for periodic gas transport in droplet microfluidics. The droplet is deionized water dyed with magenta filling ink. White dotted lines represent the initial position of the droplet movement. Scale bars: 10 mm.

The release of transport gas could drive the movement of droplets. As shown in Figure 5, when t_0_ = 0 s, the droplet was in the initial position, and SOLGM was in the closed state. As the amount of gas injection increased, the ∆*P* reached the *P*_c_, the system was in the open state, and gas was released instantaneously. As a result, the droplet moved from the position of t_0_ to t_1_. Then, due to the decrease in the system pressure and the effect of capillary force, the system was closed and the droplet was in a resting state from t_1_ to t_2_. When t_2_ = 42.4 s, the droplet completed the first periodic time movement. Therefore, the closed/open state control behavior can be switched automatically (t_3_, t_4_, t_5_, t_6_, and t_7_ in Figure 5). This pressure-driven and self-oscillating system that utilizes its gas/liquid/solid interface interaction to achieve periodic gas transport, without the need for any complex external regulations. In addition, periodic gas transport could play a promising role in the fields of microreactors [26,27], chemical reactions [28], soft robots [29,30], and fluid transports [31,32,33].

## 4. Conclusions

In summary, we have shown a new SOLGM with periodic gas transport under continuous gas injection that performs self-oscillating closed/open states. The characterization of self-oscillating behavior, the design strategy, and the parameter regulation of SOLGM have been discussed. Owing to the accumulation and compressibility of gases, the pressure of the system under a constant flow rate induces an increase. Then, when ∆*P* rises to the *P*_c_, the rapid gas release leads to a decreased pressure and reversible reconfiguration of the gating liquid. The self-oscillating behavior with periodic closed/open states for periodic gas transport control has been realized. We further demonstrate the periodic gas transport based on the porous system in droplet microfluidics. Meanwhile, the experimental results have shown that the system’s self-oscillating gas transport behavior is conditional and without the need for any complex external operational variations. The system is still developing and will be a breakthrough in many new applications, such as pneumatic actuators, multiphase microreactors, gas-involved reactions, droplet microfluidics, intelligent valves, etc.

## Data Availability

All the data are provided in the manuscript.

## Supplementary Materials

The following supporting information can be downloaded at: https://www.mdpi.com/article/10.3390/membranes12070642/s1, Video S1: Observation of SOLGM’s self-oscillating closed/open states. Here, SOLGM is a single pore (pore size of 220 µm). Then the system achieved the self-oscillating behavior for periodic gas transport control at a constant flow rate of 0.5 mL/min. Video S2: Demonstration of SOLGM for periodic gas transport in droplet microfluidics. Here, SOLGM is a porous system (pore size of 30 µm). The system realized periodic motion control of the droplet driven by the self-oscillating gas transport control at a constant flow rate of 1 mL/min.

## References

[1] Hou X., Hu Y., Grinthal A., Khan M., Aizenberg J. (2015). Liquid-based gating mechanism with tunable multiphase selectivity and antifouling behaviour. Nature.

[2] Wang S., Zhang Y., Han Y., Hou Y., Fan Y., Hou X. (2021). Design of porous membranes by liquid gating technology. Acc. Mater. Res..

[3] Zhang J., Chen B., Chen X., Hou X. (2021). Liquid-based adaptive structural materials. Adv. Mater..

[4] Sheng Z., Zhang M., Liu J., Malgaretti P., Li J., Wang S., Lv W., Zhang R., Fan Y., Zhang Y. (2021). Reconfiguring confined magnetic colloids with tunable fluid transport behavior. Natl. Sci. Rev..

[5] Zhan K., Hou X. (2018). Tunable microscale porous systems with dynamic liquid interfaces. Small.

[6] Fan Y., Sheng Z., Chen J., Pan H., Chen B., Wu F., Wang S., Chen X., Hou X. (2019). Visual chemical detection mechanism by a liquid gating system with dipole-induced interfacial molecular reconfiguration. Angew. Chem. Int. Ed..

[7] Sheng Z., Zhang J., Liu J., Zhang Y., Chen X., Hou X. (2020). Liquid-based porous membranes. Chem. Soc. Rev..

[8] Chen B., Zhang R., Hou Y., Zhang J., Chen S., Han Y., Chen X., Hou X. (2021). Light-responsive and corrosion-resistant gas valve with non-thermal effective liquid-gating positional flow control. Light Sci. Appl..

[9] Lei J., Hou Y., Wang H., Fan Y., Zhang Y., Chen B., Yu S., Hou X. (2022). Carbon dioxide chemically responsive switchable gas valves with protonation-induced liquid gating self-adaptive systems. Angew. Chem. Int. Ed..

[10] Namati E., Thiesse J., de Ryk J., McLennan G. (2008). Alveolar dynamics during respiration: Are the pores of Kohn a pathway to recruitment. Am. J. Respir. Cell Mol. Biol..

[11] Peleg O., Lim R. (2010). Converging on the function of intrinsically disordered nucleoporins in the nuclear pore complex. Biol. Chem..

[12] Stroock A., Pagay V., Zwieniecki M., Holbrook N. (2014). The physicochemical hydrodynamics of vascular plants. Annu. Rev. Fluid Mech..

[13] Friess K., Izák P., Kárászová M., Pasichnyk M., Lanč M., Nikolaeva D., Luis P., Jansen J. (2021). A review on ionic liquid gas separation membranes. Membranes.

[14] Liu J., Xu X., Lei Y., Zhang M., Sheng Z., Wang H., Cao M., Zhang J., Hou X. (2022). Liquid gating meniscus-shaped deformable magnetoelastic membranes with self-driven regulation of gas/liquid release. Adv. Mater..

[15] Yu S., Pan L., Zhang Y., Chen X., Hou X. (2021). Liquid gating technology. Pure. Appl. Chem..

[16] Zhang R., Lei J., Xu J., Fu H., Jing Y., Chen B., Hou X. (2022). Bioinspired photo-responsive liquid gating membrane. Biomimetics.

[17] Zhao L., Zhang H., Mao J., Di Y. (2022). An ICLS-based method for solving two-phase seepage free surface considering compressible gas in porous media. Comput. Geotech..

[18] Mietton-Peuchot M., Condat C., Courtois T. (1997). Use of gas-liquid porometry measurements for selection of microfiltration membranes. J. Membr. Sci..

[19] Sheng Z., Wang H., Tang Y., Wang M., Huang L., Min L., Meng H., Chen S., Jiang L., Hou X. (2018). Liquid gating elastomeric porous system with dynamically controllable gas/liquid transport. Sci. Adv..

[20] Butt H., Graf K., Kappl M. (2003). Physics and Chemistry of Interfaces.

[21] Raturi P., Singh J. (2019). An intelligent dual mode filtration device for separation of immiscible oil/water mixtures and emulsions. Appl. Surf. Sci..

[22] Qu M., Abdelaziz O., Gao Z., Yin H. (2018). Isothermal membrane-based air dehumidification: A comprehensive review. Renew. Sust. Energ. Rev..

[23] Bazyar H., Javadpour S., Lammertink R. (2016). On the gating mechanism of slippery liquid infused porous membranes. Adv. Mater. Interfaces.

[24] Bazyar H., Lv P., Wood J., Porada S., Lohse D., Lammertink R. (2018). Liquid–liquid displacement in slippery liquid-infused membranes (SLIMs). Soft Matter.

[25] Zhang R., Gao C., Tian L., Wang R., Hong J., Gao M., Gui L. (2021). Dynamic pneumatic rails enabled microdroplet manipulation. Lab Chip.

[26] Hone C., Kappe C. (2020). Membrane microreactors for the on-demand generation, separation, and reaction of gases. Chemistry.

[27] Liu Y., Chen G., Yue J. (2020). Manipulation of gas-liquid-liquid systems in continuous flow microreactors for efficient reaction processes. J. Flow. Chem..

[28] Liu W., Wang M., Sheng Z., Zhang Y., Wang S., Qiao L., Hou Y., Zhang M., Chen X., Hou X. (2019). Mobile liquid gating membrane system for smart piston and valve applications. Ind. Eng. Chem. Res..

[29] Drotman D., Jadhav S., Sharp D., Chan C., Tolley M.T. (2021). Electronics-free pneumatic circuits for controlling soft-legged robots. Sci. Robot..

[30] Belding L., Baytekin B., Baytekin H., Rothemund P., Verma M., Nemiroski A., Sameoto D., Grzybowski B., Whitesides G. (2018). Slit tubes for semisoft pneumatic actuators. Adv. Mater..

[31] Akhmetshina A., Davletbaeva I., Grebenschikova E., Sazanova T., Petukhov A., Atlaskin A., Razov E., Zaripov I., Martins C., Neves L. (2016). The effect of microporous polymeric support modification on surface and gas transport properties of supported ionic liquid membranes. Membranes.

[32] Han Y., Zhang Y., Zhang M., Chen B., Chen X., Hou X. (2021). Photothermally induced liquid gate with navigation control of the fluid transport. Fundam. Res..

[33] Lv W., Sheng Z., Zhu Y., Liu J., Lei Y., Zhang R., Chen X., Hou X. (2020). Highly stretchable and reliable graphene oxide-reinforced liquid gating membranes for tunable gas/liquid transport. Microsyst. Nanoeng..

